# Type VI secretion system of *Pseudomonas aeruginosa* is associated with biofilm formation but not environmental adaptation

**DOI:** 10.1002/mbo3.991

**Published:** 2020-01-21

**Authors:** Lihua Chen, Yaru Zou, Asmaa Abbas Kronfl, Yong Wu

**Affiliations:** ^1^ Department of Clinical Laboratory The Third Xiangya Hospital Central South University Changsha China

**Keywords:** biofilm formation, *clpV1*, environmental adaptation, *Pseudomonas aeruginosa*, Type VI secretion system

## Abstract

*Pseudomonas aeruginosa* encodes three type VI secretion systems (T6SSs), namely H1‐, H2‐, and H3‐T6SS. *P. aeruginosa* hemolysin‐coregulated protein (Hcp) is the effector protein and the hallmark of T6SS. Although T6SS is ubiquitous and affects ecology and human health, its general mechanism and physiological role are still not fully understood. Therefore, in this study, we investigated the impact of the *P. aeruginosa* T6SS on biofilm formation and environmental adaptation. To this end, we collected *P. aeruginosa* clinical isolates, divided them into strong biofilm formation (SBF) and nonbiofilm formation (NBF) groups based on their biofilm‐forming ability, and compared their associated clinical characteristics. The duration of hospitalization was longer in patients infected with SBF than those infected with NBF strains. The expression levels of T6SS‐related genes (*hcp1* and *hcp3*) and a quorum‐sensing gene (*lasR*) were higher in the SBF group as compared to those in the NBF group. In addition, the expression level of *lasR* was negatively associated with that of *hcp1*, but was positively associated with those of *hcp2* and *hcp3*. Moreover, we evaluated the expression of T6SS‐ and biofilm‐associated genes in planktonic and biofilm cells of the *P. aeruginosa* strain PAO1, and constructed strain PAO1△*clpV1* to study the adaptation characteristics of H1‐T6SS. The expression levels of *hcp1, hcp2, hcp3, lasR,* and other biofilm‐associated genes were significantly higher in PAO1 biofilm cells as compared to those of planktonic cells. However, except for swarming ability as a vital feature for biofilm formation, there were no significant differences in the biofilm‐forming ability and expression of biofilm‐associated genes, adherence ability, growth characteristics, resistance to acid and osmotic pressure, surface structure, and morphology between the PAO1△*clpV1* and PAO1 wild‐type strains. Collectively, our results suggest that T6SS might play a role in biofilm formation and that H1‐T6SS does not contribute to environmental adaptation in *P. aeruginosa*.

## Graphical abstract

Our findings showed that in clinical isolates of Pseudomonas aeruginosa with strong biofilm‐forming group, the expression levels of the T6SS‐related genes hcp1 and hcp3 were significantly higher than those in nonbiofilm‐forming group, and all three T6SS‐related genes (hcp1, hcp2, and hcp3) were significantly more strongly expressed in biofilm cells than in the planktonic form of the type strain PAO1. A H1‐T6SS mutant strain had no impact on biofilm formation or environmental adaptation ability, but swarming motility was reduced, which is another important aspect of biofilm formation ability. Our results suggest that T6SS is associated with biofilm formation but not environmental adaptation.

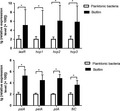

## 1 INTRODUCTION

*Pseudomonas aeruginosa* is an opportunistic gram‐negative pathogen that is widely distributed in diverse environments. It is a common source of hospital‐acquired infections. Indeed, *P. aeruginosa* is responsible for a wide range of severe acute and chronic infections. It is the most common pathogen detected in the respiratory tract of patients with cystic fibrosis, which is a leading cause of morbidity and mortality (Kordes et al., 2019). The data from CHINET showed that among 22,774–190,610 clinical isolates collected from 8 to 34 hospitals throughout China from 2005 to 2017, *P. aeruginosa* was one of the four common isolates (Zheng, Li, Zhang, & Pei, 2019).

Bacteria generally exist in two major forms, namely planktonic cells and biofilm cells. A biofilm is a structured and complex community of microorganisms that adheres to biotic or abiotic surfaces and is surrounded by a complex matrix of extracellular polymeric substances (Chen et al., 2016). In biofilms, the bacteria communicate with each other to share nutrients, signals, and genetic information, and offer mutual protection from desiccation, predation, and antibiotics. Biofilms pose a serious challenge to humanity, as these cells are 10‐ to 1000‐fold more resistant to antibacterial agents than isolated planktonic cells (Hoiby, 2017). *P. aeruginosa* is one of the most frequently detected bacteria that cause biofilm‐associated infections (Del, 2018). Cystic fibrosis, chronic bronchitis, bronchiectasis, and ventilator‐associated pneumonia are the most common biofilm‐associated diseases caused by *P. aeruginosa*. Moreover, biofilm formation contributes to the wide distribution of *P. aeruginosa* in diverse environments*.*

Bacterial cells have developed multiple tactics to communicate with their surrounding environment, including a specialized secretion system. In particular, the type VI secretion system (T6SS) is a recently described secretion system that is present in nearly 25% of gram‐negative bacteria, including *P. aeruginosa*, *Vibrio cholerae*, *Escherichia coli*, and *Acinetobacter* spp*.* However, the current knowledge about the general mechanism and physiological role of T6SS is limited and requires further study (Del, 2018). In addition, the T6SS has attracted wide attention in recent years owing to its presence in various pathogenic bacteria and recognition of its ability to interfere with the ecosystem and human health (Gao, Mu, Qin, Sun, & Cui, 2017).

T6SS is typically encoded by a group of tightly regulated gene clusters with 13 conserved genes as the core components. This system is believed to resemble the contractile tails of bacteriophages. Hemolysin‐coregulated protein (Hcp) forms hexameric rings similar to the phage lambda gpV tail tube protein. Valine–glycine repeat protein G (VgrG) shares structural similarities with the spike complex of the T4 bacteriophage. Hcp and VgrG can be found in the culture supernatants of most bacteria containing T6SS gene clusters. Notably, Hcp is considered as the hallmark of T6SS and can be used as a biomarker for activation of T6SS (Wang et al., 2018). In this bacteriophage‐like structure, ClpV protein acts as an AAA + ATPase by providing energy to the system. Loss of *clpV1* in *P. aeruginosa*, or even loss of its ATPase motif, is sufficient to completely abrogate the secretion function (Bingle, Bailey, & Pallen, 2008; Hachani et al., 2011).

*P. aeruginosa* is a common model organism to study T6SS because it harbors three Hcp secretion islands, namely H1‐T6SS, H2‐T6SS, and H3‐T6SS. The secreted effector protein of H1‐T6SS is Hcp1, the secretion of which is facilitated by ClpV1. The three T6SSs of *P. aeruginosa* also appear to have distinct functions. H1‐T6SS targets prokaryotic cells and contributes to the survival advantage of *P. aeruginosa* (Boulant et al., 2018). H2‐T6SS and H3‐T6SS target not only prokaryotic but also eukaryotic cells (Sana, Berni, & Bleves, 2016).

T6SS is associated with several essential phenotypes of bacteria, including biofilm formation and environmental adaptation. However, only few studies have compared the expression of T6SS among *P. aeruginosa* clinical isolates with different biofilm‐forming abilities. Moreover, further research is needed to clarify whether H1‐T6SS plays a role in the environmental adaptation of *P. aeruginosa*, contributing to its apparent survival advantage under diverse conditions.

To resolve these questions, in the present study, we collected *P. aeruginosa* clinical isolates and investigated the possible roles of T6SS in biofilm formation. We further used the *P. aeruginosa* model strain PAO1 to study the specific effects of H1‐T6SS on the biofilm‐forming ability and environmental adaptation of *P. aeruginosa.* Our findings would support the theory to consider T6SS as a therapeutic target of treatments for chronic *P.aeruginosa* infections.

## 2 MATERIALS AND METHODS

### 2.1 Strains and growth conditions

The *P.aeruginosa* laboratory strain PAO1 was a generous gift by Professor Mingqiang Qiao of Nankai University, Tianjing, China. The clinical strains used in this study were isolated from the sputum of patients suffering from ventilator‐associated pneumonia and chronic bronchitis between November 2014 and March 2015 at the Third Xiangya Hospital of Central South University, Changsha, China.

The bacteria were inoculated on blood agar and incubated at 37°C for 18–24 hr. The colonies were subsequently inoculated into skim milk and preserved at −20°C until further use.

### 2.2 Preparation of biofilm and planktonic cells of PAO1

The *P. aeruginosa* strain PAO1 was inoculated on blood agar and incubated for 18 hr at 37°C. A single bacterial colony was picked up with a sterile wire loop and inoculated in 5 ml of Luria–Bertani (LB) broth, followed by incubation at 37°C for 18 hr with vigorous agitation at 180 rpm. The obtained cells were used in the assessment of the planktonic form.

In order to obtain a biofilm of PAO1, the cells of the planktonic form were centrifuged, and the supernatant was discarded. Normal saline was added to the bacterial precipitate and adjusted to 0.5 McFarland standard. The bacteria were then seeded in a 6‐well cell culture plate (Corning Costar) at 800 μl per well with 2.4 ml LB broth and incubated at 37°C without agitation. After every 48 hr, the LB broth was discarded and finally replaced with fresh LB for 6 days. After 6‐day incubation, the LB broth was discarded and the plates were gently washed three times with 1X phosphate‐buffered saline (PBS, pH 7.4). The cells adhered to the wells were scraped down with a cell scraper and collected together as the biofilm of PAO1 strain.

### 2.3 Quantification of the biofilm‐forming ability of *P. aeruginosa* clinical strains

The clinical strains of *P. aeruginosa* were inoculated on blood agar and incubated at 37°C for 18–24 hr. A single bacterial colony was picked up and inoculated in 5 ml of LB broth, followed by overnight incubation at 37°C with vigorous agitation (180 rpm). The culture was then centrifuged, and the supernatant was discarded. Then, normal saline was added to the bacterial precipitate and adjusted to 0.5 McFarland standard. Each well of the sterile 96‐well polystyrene microtiter plate was filled with 50 μl of culture and 150 μl of fresh LB broth. Negative control wells contained only LB broth. The plates were aerobically incubated at 37°C without agitation. After every 24 hr, the LB broth was discarded and replaced with fresh LB for 72 hr. The LB broth was discarded, and the plates were gently washed three times with 1X PBS (pH 7.4) and stained with 200 μl of 0.1% crystal violet (Sigma‐Aldrich) for 15 min at room temperature (approximately 25°C). The biofilm‐forming ability was quantified by measuring the corresponding optical density of the supernatant at 570 nm (OD_570_) following the solubilization of crystal violet in 95% ethanol.

Based on the OD shown by the bacterial films, the tested clinical bacterial strains were classified into two groups, (a) nonbiofilm‐forming group (NBF), in which the OD was less than or equal to the mean value of the control wells (ODc), and (b) strong biofilm‐forming group (SBF), in which the OD was higher than 4‐fold of the ODc (Stepanovic, Vukovic, Dakic, Savic, & Svabic‐Vlahovic, 2000).

### 2.4 Construction of the PAO1 *clpV1* gene deletion mutant (MT) strain

To specify the role of T6SS, the PAO1 *clpV1* gene was deleted by homologous recombination, using the vector plasmids pUC19 and pJQ200SK and the suicide plasmid Pcvd442 (BGI Genomics). The homologous recombination procedure was performed as previously described (Choi & Schweizer, 2005). Gene deletion was verified by polymerase chain reaction (PCR) with *clpV1* internal and external primers. The deletion MT strain was cultured as described above for wild‐type (WT) PAO1 strain.

### 2.5 RNA isolation and cDNA synthesis

For PAO1 strain, the planktonic cells and biofilm were prepared as described above. Then, 1.5 ml of the cells were collected and transferred to a sterilized microcentrifuge tube for RNA extraction.

For the clinical isolates, a single colony from overnight blood agar culture was inoculated in 5 ml LB followed by incubation at 37°C, 180 rpm for 16–18 hr. Subsequently, 1 ml of this culture was centrifuged, and the cell pellet was used for RNA extraction.

RNA extraction was performed using the E.Z.N.A Total RNA Kit (Omega Bio‐tek) according to the manufacturer's instructions. Then, 1 μg of total RNA was used to synthesize cDNA using the EasyScript First‐Strand cDNA Synthesis SuperMix Kit (TransGen Biotech). The synthesized cDNA was preserved at −20°C before proceeding to quantitative PCR (qPCR).

### 2.6 Measurement of gene expression levels by qPCR

After cDNA synthesis, qPCR was performed to determine the expression levels of biofilm‐ and secretion‐related genes *hcp1*, *hcp2*, *hcp3*, *lasR*, *pslA*, *pelA*, *pilA*, and *fliC* using the TransStart SYBR Green qPCR SuperMix UDG Kit (TransGen Biotech) and the Eppendorf Real‐Time system (Eppendorf). Primers (Sangon Biotech) used for qPCR are listed in Table 1. A control lacking the template was included in the analysis as a negative control. For quantification, 16S rRNA was used as a marker gene. Relative expression levels were determined using the 2^−ΔΔCt^ method. Reactions were prepared according to the manufacturer's instructions with 10‐fold dilution of cDNA as the template. All reactions were carried out in triplicates with at least two biological replicates.

**Table 1:** Oligonucleotides used in this study

Primer name	Primer sequence (5′‐3')
For real‐time PCR	
*hcp1*‐F	CGAGAACGTGACCCTGAACT
*hcp1*‐R	TGTTCCAGCCGTACTTGACC
*hcp2*‐F	CGGTGGTCATCACCAAGGTC
*hcp2*‐R	CTGGCAGTTGTGCATGTAGTCC
*hcp3*‐F	GGATGCGATCATTCTCGATT
*hcp3*‐R	GGTCGAGGTGTCGATGAACT
*lasR*‐F	TCGGTTATCTGCAACTGCTC
*lasR*‐R	GACCCAAATTAACGGCCATA
*pslA*‐F	GGAACAGCCAGGTAATGGAC
*pslA*‐R	TCCAGGGTATCGAGGAACAG
*pelA*‐F	GGAACAGCCAGGTAATGGAC
*pelA*‐R	TCCAGGGTATCGAGGAACAG
*pilA*‐F	GATCAACCCGCTGAAGACC
*pilA*‐R	TGTTTCGGTCGCAGTAGAAG
*fliC*‐F	CGACAAGGGTGTACTGACCA
*fliC*‐R	GACCTTCACTGCGACCTGAC
*16SrRNA*‐F	AACGCGAAGAACCTTACC
*16SrRNA*‐R	AAGGGTTGCGCTCGTTAC
For isogenic deletion mutant	
Up‐*clpv1*‐F	ATAGAATTCTAGAGCCGTCCGAACCTCTGAACCTGCTC EcoRI‐XbaI
Up‐*clpv1*‐R	ATAGGATCCACTGATCTCACTCATGTTCCTTCTCCTTGTGCTCG BamHI
Down‐*clpv1*‐F	ATAGGATCCGACTTCGCCGAGGCCGAGTGACAAC BamHI
Down‐*clpv1*‐R	TATAAGCTTCTAGATATTCGGCGCCGACCACCAGGTACTC XbaI‐HindIII
*clpV1*‐In‐F	CAAGCGCTTTACCGTGGACCTCAC
*clpV1*‐In‐R	GTCGAGACGCACCTCGCCTTC
*clpV1*‐out‐F	CTGTGTTCGAGCGCTTCCTGGCACGCTACGTGTC
*clpV1*‐out‐R	GGAGATATTGATGGTGCCGTCGGCGTTGAACTC

### 2.7 Adaptation characteristics of *P. aeruginosa* H1‐T6SS

To assess the influence of H1‐T6SS on growth, single colonies of the PAO1 WT and MT strains from overnight blood agar cultures were inoculated in 5 ml LB and cultured at 37°C, 180 rpm for 16–18 hr. The overnight solutions were diluted to 2 McFarland standard, and 150 μl of the diluted solution was added to 10 ml LB and cultured at 37°C, 180 rpm. Starting from 0 hr, the OD_630_ was measured after every 2 hr up to 24 hr to construct the growth curve.

The influence of H1‐T6SS on biofilm formation was tested in a 96‐well cell culture microtiter plate with crystal violet staining as described in Section 2.3.

The adherence abilities of the WT and MT cells were compared between the 100‐fold diluted overnight solutions; 200 μl of the diluted solution was added to each well of the 96‐well plate and cultured at 37°C for 2 hr under static conditions. The supernatant was removed, and the wells were washed gently to remove any nonadherent bacteria. The adherent bacteria were then stained with crystal violet as described in Section 2.3 and the OD_570_ values were measured.

Resistance to acid stress was measured by adjusting the pH of the LB broth to 4.6, 4.8, and 5.0 with 1 N NaOH and HCl. The growth characteristics of PAO1 WT and MT strains were compared at the above‐mentioned pH values.

To assess the resistance to high osmotic pressure, the overnight cultures of PAO1 WT and MT were adjusted to OD_630_ = 0.5 with fresh LB broth. Then, 2 ml of the diluted cultures was mixed with 2 ml of LB medium containing 3 M NaCl and cultured at 37°C, 180 rpm. Starting from 0 hr, the bacterial survival rates were measured every hour to 4 hr by the plate culture method.

The swarming motility was assessed as previously described (She et al., 2018). The swarming agar contained 0.5% (w/v) Difco Bacto agar and 0.5% glucose in nutrient broth. A quantity of 5 μl of diluted bacterial cultures was spotted at the center of the agar followed by incubation at 37°C under static conditions. After incubation at 37°C for 20 hr, photographs of the plates were taken to measure the diameter of swarming zone.

Finally, the morphological changes between PAO1 WT and PAO1 MT strains were analyzed using a scanning electron microscope.

### 2.8 Statistical analysis

Data are expressed as means ± standard deviation for continuous variables and as frequencies for categorical variables. Differences between groups were assessed using chi‐square statistics for categorical variables. Continuous variables with normal and non‐normal distributions were compared using Student's *t* test and Mann–Whitney *U* test, respectively. A *p* < .05 was considered statistically significant. Pearson's correlation analysis was performed using significant variables to assess correlations between continuous variables. Statistical analysis was performed using SPSS version 22.0 statistical software (SPSS Inc.).

## 3 RESULTS

### 3.1 Baseline characteristics and gene expression profiles of the clinical strains

**Figure 1:**
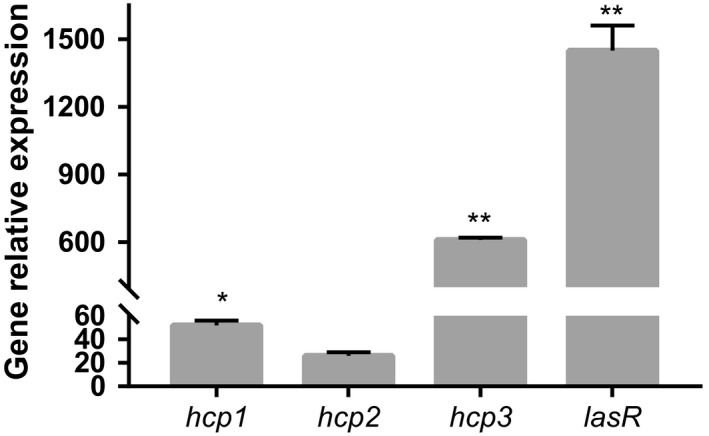
The mRNA levels were measured by real‐time PCR. The ratios of *hcp1*, *hcp2*, *hcp3*, and *lasR* expression levels in strong biofilm formation strains to those in nonbiofilm formation strains are shown.*p < .05 and **p < .01

In this study, 46 clinical strains were collected and divided into the SBF and NBF groups according to their biofilm‐forming abilities. The SBF isolates were associated with longer duration of hospitalization (Table 2) and showed significantly higher expression levels of T6SS‐associated genes and *lasR* compared to those in the NBF group (Figure 1).

**Table 2:** Baseline characteristics of the *Pseudomonas aeruginosa* clinical isolates

	Strong biofilm formation group (*n* = 23)	Nonbiofilm formation group (*n* = 23)	*p* value
Duration in days	38.4783 ± 12.64505	8.4783 ± 3.65401	<.001
OD_570_	1.8362 ± 0.69635	0.401 ± 0.08029	<.001
Male	14 (60.87%)	15 (65.22%)	.948
Female	9 (39.13%)	8 (34.78%)	.761
Age (year)	65.45 ± 11.32	60.05 ± 10.09	.225

### 3.2 Correlation between the quorum‐sensing gene *lasR* and T6SS‐related genes in the SBF strains

**Figure 2:**
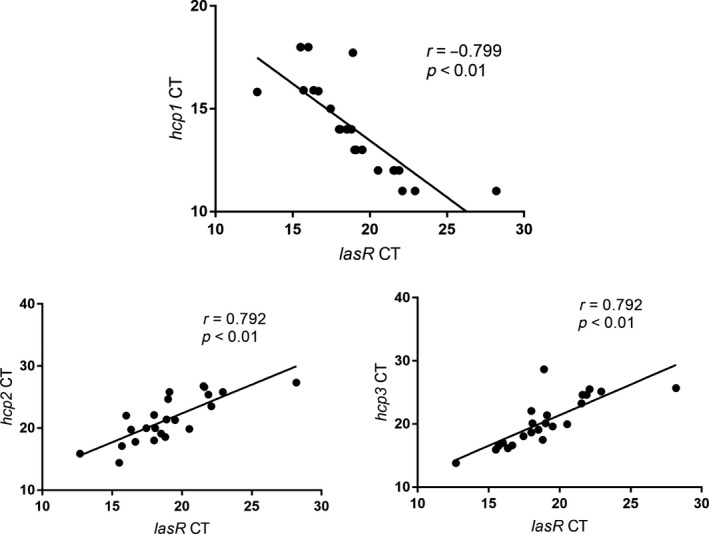
Pearson correlation coefficients for TSSS‐ and quorum sensing‐related genes in the strong biofilm formation strains

Pearson's correlation analysis was used to assess the correlations between quorum‐sensing *lasR* and T6SS‐associated genes (*hcp1*, *hcp2*, and *hcp3*) in the SBF clinical strains. As shown in Figure 2, there was a strong negative correlation between *lasR* and T6SS *hcp1* levels, suggesting that *lasR* negatively regulates the expression of *hcp1*. In contrast, *lasR* levels positively correlated with *hcp2* and *hcp3* levels.

### 3.3 Expression levels of biofilm‐ and secretion‐related genes in PAO1 biofilm and planktonic cells

**Figure 3:**
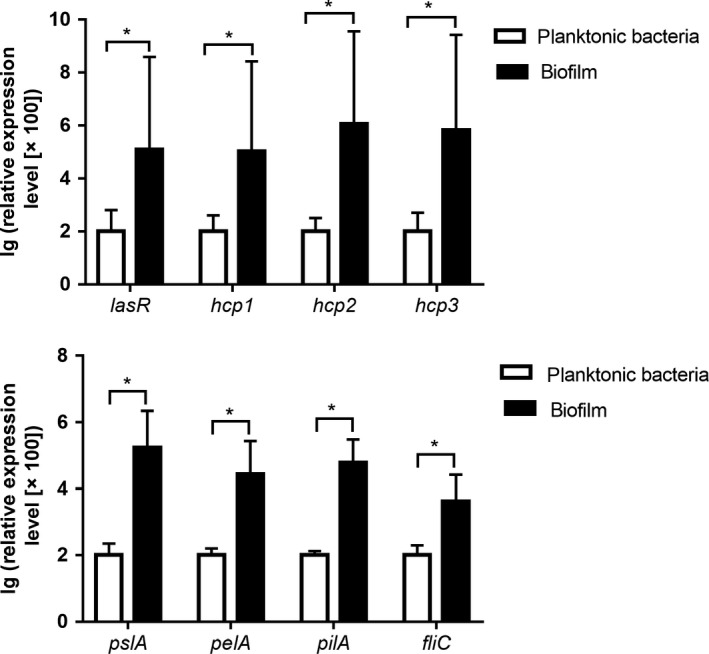
Relative expression levels of *hcp1‐3*, *lasR*, *pslA*, *pelA*, *pilA*, and *fliC* in PAO1 biofilm and planktonic cells. *p < .05

As shown in Figure 3, the expression levels of *hcp1–3*, *lasR*, *pslA*, *pelA*, *pilA,* and *fliC* were significantly higher in the PAO1 biofilm than those in the planktonic cells. Specifically, the expression levels of *hcp1*, *hcp2*, *hcp3*, *lasR*, *pslA*, *pelA*, *pilA,* and *fliC* in the biofilm were 1,226‐, 1,045‐, 11,268‐, 6,654‐, 1,714‐, 274‐, 604‐, and 42‐fold higher than those in the planktonic cells, respectively.

### 3.4 Adaptation characteristics of PAO1 MT and WT strains

**Figure 4:**
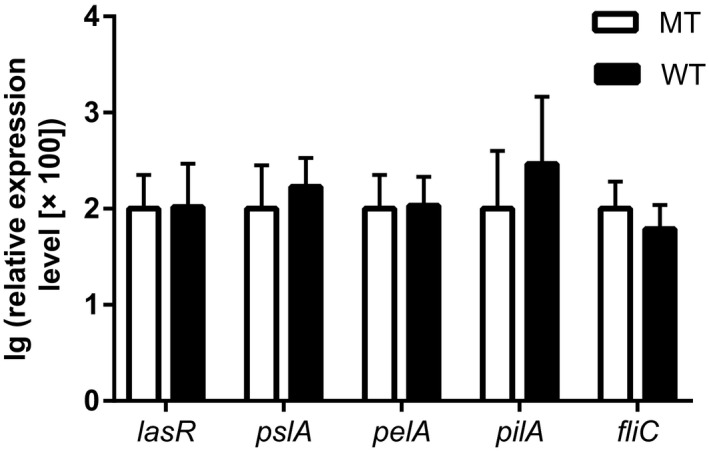
Gene expression levels in PAO1 WT and PAO1 MT strains

Using a homologous recombination technique, *clpV1* was successfully deleted in the PAO1 strain as verified by the lack of *clpV1* amplification in the MT strain by PCR. The expression levels of *lasR*, *pslA*, *pelA*, *pilA*, and *fliC* in the PAO1 WT showed 1.04‐, 1.69‐, 1.08‐, 2.9‐, and 0.61‐fold difference compared to those of the MT strain, with no significant differences between the two strains (*p* > .05) as shown in Figure 4.

**Figure 5:**
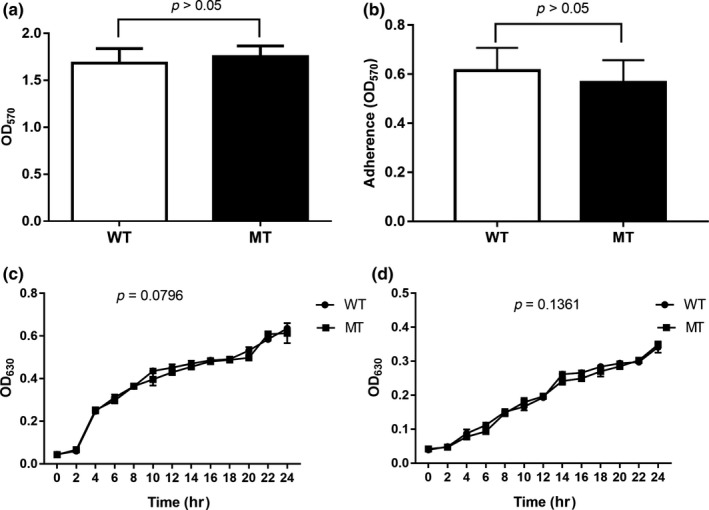
Comparison of the biofilm‐forming ability (a), adherence ability (b), growth curve at 37°C (c), and growth curve at 42°C (d) between PAO1 WT and PAO1 MT strains

**Figure 6:**
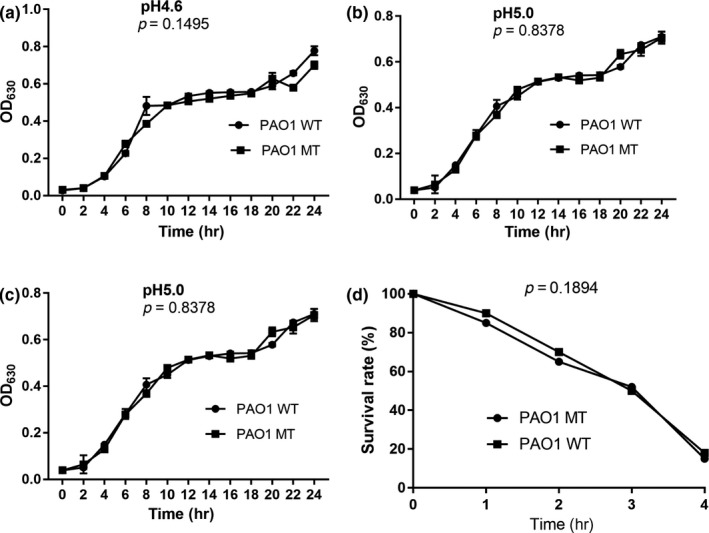
Comparison of resistance to acid (a, b, c) and high osmotic pressure (d) between PAO1 WT and PAO1 MT strains

**Figure 7:**
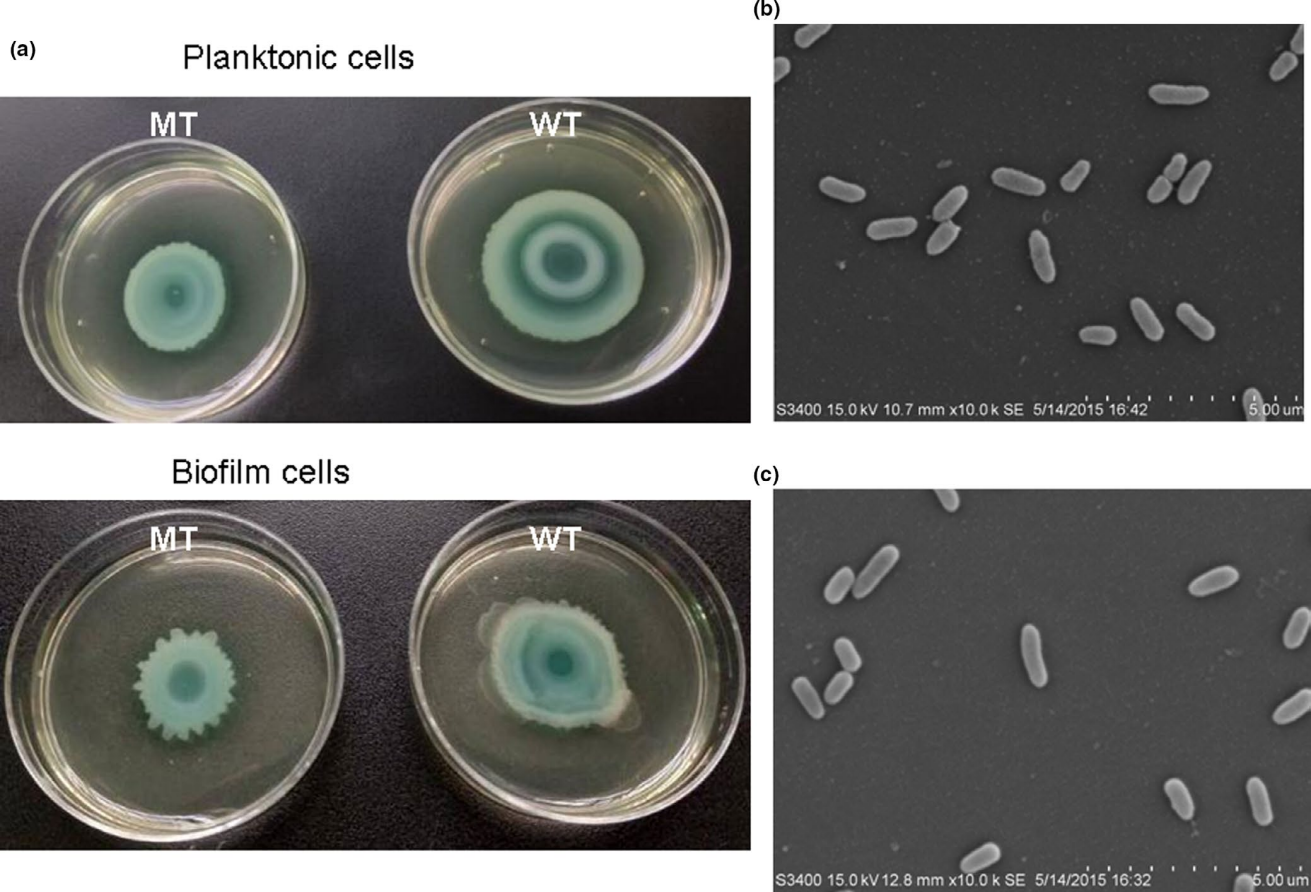
Swarming motility (a) and morphological features of the PAO1 WT (b) and PAO1 MT (c) strains analyzed by scanning electron microscopy

There were no significant differences between the PAO1 WT and MT strains in the adaptation characteristics, including growth characteristics at 37°C and 42°C, and resistance to acid and osmotic pressure as shown in Figures 5 and 6, respectively. In addition, the deletion of *clpV1* did not influence the biofilm‐forming ability and adherence ability of the PAO1 strain. Further, scanning electron microscopy showed no differences in the length and surface structure between the two strains. However, the PAO1 WT strain showed better swarming motility in both biofilm and planktonic cells (Figure 7).

## 4 DISCUSSION

Biofilm‐forming ability is one of the primary causes of the resistance of *P. aeruginosa* to antibiotics, leading to chronic infections. In this study, we observed that the SBF group‐infected patients showed longer hospitalization as compared to the NBF group‐infected patients. Although T6SS is reportedly associated with biofilm formation, the available information on the relationship between T6SS and biofilm‐forming ability of *P. aeruginosa* clinical isolates is limited. The present study showed that the expression levels of the T6SS‐related genes *hcp1* and *hcp3*, and the quorum sensing‐related gene *lasR* were significantly higher in the SBF group as compared to those in the NBF group. However, there was no difference in the expression level of *hcp2*.

T6SS of *P. aeruginosa* was first described by Mougous (2006), who reported that the level of Hcp1 antibody was higher in the sera of patients with long‐term cystic fibrosis (more than 10 years) than in the cases of short‐term infection (0.3–4 years), which confirmed the role of H1‐T6SS in chronic *P. aeruginosa* infections. Subsequently, Zhang, Hinz, Nadeau, and Mah (2011) showed that the expression level of *hcp1* in *P. aeruginosa* strain PA14 was significantly higher in biofilm than in planktonic cells. H2‐T6SS was also reported to be responsible for severe acute infections but not chronic infections (Boulant et al., 2018). These previous findings explain the lack of difference in *hcp2* expression between the SBF and NBF groups. In contrast to H1‐T6SS, the functions of H3‐T6SS of *P. aeruginosa* are poorly understood. Only one report showed that icmF3, a component of H3‐T6SS, plays critical roles in environmental adaptation and pathogenesis of *P. aeruginosa* PAO1 (Lin et al., 2015). Our results also suggest that H3‐T6SS might be associated with biofilm formation by *P. aeruginosa.* The biofilm of *P. aeruginosa* PAO1 strain showed significantly higher expression of all three T6SS‐related genes (*hcp1*, *hcp2*, and *hcp3*) than those in planktonic cells, which further confirms the association between T6SS and biofilm formation.

Quorum sensing, a bacterial cell–cell communication system, plays a critical role in regulating the expression of virulence genes and biofilm formation in *P. aeruginosa. LasR* is one of the most important quorum sensing‐related genes in *P. aeruginosa* (Ahmed & Salih, 2019; Lee & Zhang, 2015)*.* Quorum sensing has also been reported to regulate the three T6SSs of *P. aeruginosa* in the strains PAO1 and PA14 (Pena et al., 2019; Sana et al., 2012). In this study, both the SBF clinical isolates and biofilm of *P. aeruginosa* PAO1 showed significantly higher expression level of *lasR* compared to those in the NBF and PAO1 planktonic cells. This finding is consistent with several reports demonstrating that inhibiting quorum sensing might be a useful target for suppressing biofilm formation (Pattnaik et al., 2018; Taha, Saafan, Ahmedy, El, & Khairalla, 2019). Lesic, Starkey, He, Hazan, and Rahme (2009) reported that quorum sensing in *P. aeruginosa* negatively regulated the expression of H1‐T6SS but positively regulated the expression of H2‐T6SS and H3‐T6SS. Therefore, the present results showing the correlations between the three T6SSs and *lasR* are consistent with the above results.

Extracellular polysaccharides are the main components of the biofilm matrix that protect the bacteria from host immune response and antibiotics (Al‐Wrafy, Brzozowska, Gorska, & Gamian, 2017). Pel and Psl are two types of extracellular polysaccharides found in nonmucoid strains of *P. aeruginosa* (Di Martino, 2018; RyderByrd & Wozniak, 2007). Our results showed that in PAO1 biofilms, in addition to *pel* and *psl*, the expression levels of *pilA* (encoding type IV pili) and *fliC* were significantly higher than those in planktonic cells. PilA, a major subunit of pilin, plays a vital role in pilus assembly and is involved in attachment and motility, whereas FliC is essential for flagellum assembly and motility in *P. aeruginosa* (Nguyen et al., 2018). Adherence ability and motility are also essential properties for biofilm development, and therefore, flagella play an important role in the adherence of *P. aeruginosa*. Moreover, *P. aeruginosa* lacking type IV pili could only form a single layer but not the mature mushroom‐shaped biofilm structure (Barken et al., 2008; Klausen, Aaes‐Jorgensen, Molin, & Tolker‐Nielsen, 2003).

Hcp is one of the two types of proteins secreted by T6SS, and its secretion requires the energy from ClpV. Therefore, to verify the functions of H1‐T6SS, we deleted the *clpV1* gene of *P. aeruginosa* PAO1. However, the deletion did not affect the strain's biofilm formation ability and other adaptation characteristics (adherence ability, growth characteristics, resistance to acid and osmotic pressure, bacterial surface structure, and morphology), and the expression levels of biofilm‐associated genes (*lasR*, *pslA*, *pelA*, *pilA*, and *fliC*) were consistent with the observed phenotypes. However, swarming motility, another important criterion for the biofilm formation by *P. aeruginosa* PAO1 (Chatterjee et al., 2017), was inhibited by the deletion of *clpV1*. These findings are in alignment with the previous studies. For instance, Gallique et al. (2017) reported that mutation in *hcp1*, *hcp2*, or *hcp3* of *P. fluorescens* MFE01 could not reduce its biofilm formation, but these three Hcp proteins were essential for the formation of mature biofilm structure. Lin et al. (2015) also showed that deletion of *icmF3* (a component of H3‐T6SS) in *P. aeruginosa* PAO1 did not affect its growth in LB at 37°C, whereas *Vibrio anguillarum* T6SS was found to facilitate resistance to stresses such as ethanol and low pH (Weber, Hasic, Chen, Wai, & Milton, 2009). Another report showed that *Yersinia pseudotuberculosis* T6SS was involved in the resistance to osmotic and acidic stress (Zhang et al., 2013). These discrepancies among studies maybe due to the species‐specific differences, along with differences in their typical habitats.

It must be noted that T6SS is not only regulated at the transcriptional level, but is also subjected to specific post‐transcriptional and post‐translational regulation. For example, the post‐transcriptional regulator RsmA represses three T6SSs of *P. aeruginosa* (Allsopp et al., 2017). At the post‐translational level, the activity of T6SS is tightly regulated by a threonine kinase/phosphatase pair (PpkA/PppA) pathway, which also requires participation of TagT, S, R, and Q (Casabona et al., 2013). Ideally, the activity of T6SS can be analyzed by Western blot. However, the experiment could not be performed due to unavailability of the required antibodies. This limitation of the study prevents us from confirming the inconsistency between mRNA levels and T6SS activity. The other limitation of this study is the lack of complementation analyses with Δ*clpV1* strain to verify the roles of H1‐T6SS. In addition, to avoid the compensatory action of H2‐T6SS and H3‐T6SS, it would be ideal to spontaneously delete H2‐ and H3‐T6SS to study the specific effects of H1‐T6SS.

## 5 CONCLUSIONS

Overall, our findings suggest that T6SS contributes to the biofilm formation by *P. aeruginosa*. Quorum sensing also appears to regulate T6SS to some extent. H1‐T6SS does not influence the adaptability of *P. aeruginosa* but plays a critical role in swarming motility, which is another important criterion for the biofilm formation by *P. aeruginosa.* Our findings highlight the potential of T6SS as a therapeutic target for treatment of biofilm‐associated diseases caused by *P. aeruginosa*.

## CONFLICT OF INTEREST

None declared.

## AUTHOR CONTRIBUTIONS

Lihua Chen: Conceptualization; Funding acquisition; Resources; Writing‐original draft‐Lead; Writing‐review & editing. Yaru Zou: Data curation; Formal analysis; Investigation; Validation‐Lead; Writing‐review & editing. Asmaa Abbas Kronfl: Data curation; Formal analysis; Investigation; Writing‐review & editing. Yong Wu: Conceptualization; Funding acquisition; Project administration‐Lead; Resources; Supervision‐Lead; Writing‐original draft; Writing‐review & editing.

## ETHICS STATEMENT

All procedures performed in this study were conducted according to the ethical standards of the institutional research committee. The study protocol was approved by the IRB of the Third Xiangya Hospital, Central South University (No. 2014‐S024). In addition, informed consent was obtained from the patients.

## Data Availability

All data generated or analyzed during this study are included in this published article.
